# Radio Channel Capacity with Directivity Control of Antenna Beams in Multipath Propagation Environment

**DOI:** 10.3390/s21248296

**Published:** 2021-12-11

**Authors:** Cezary Ziółkowski, Jan M. Kelner, Jarosław Krygier, Aniruddha Chandra, Aleš Prokeš

**Affiliations:** 1 Institute of Communications Systems, Faculty of Electronics, Military University of Technology, 00908 Warsaw, Poland; cezary.ziolkowski@wat.edu.pl (C.Z.); jaroslaw.krygier@wat.edu.pl (J.K.); 2 Department of Electronics and Communication Engineering, National Institute of Technology, Durgapur 713209, India; aniruddha.chandra@ieee.org; 3 Department of Radio Electronics, Brno University of Technology, 61600 Brno, Czech Republic; prokes@vutbr.cz

**Keywords:** wireless communications, radio propagation, multi-elliptical propagation model, directional antennas, radio channel capacity, beam misalignment, non-line-of-sight (NLOS) conditions

## Abstract

The basic technology that will determine the expansion of the technical capabilities of fifth generation cellular systems is a massive multiple-input-multiple-output. Therefore, assessing the influence of the antenna beam orientations on the radio channel capacity is very significant. In this case, the effects of mismatching the antenna beam directions are crucial. In this paper, the methodology for evaluating changes in the received signal power level due to beam misalignment for the transmitting and receiving antenna systems is presented. The quantitative assessment of this issue is presented based on simulation studies carried out for an exemplary propagation scenario. For non-line-of-sight (NLOS) conditions, it is shown that the optimal selection of the transmitting and receiving beam directions may ensure an increase in the level of the received signal by several decibels in relation to the coaxial position of the beams. The developed methodology makes it possible to analyze changes in the radio channel capacity versus the signal-to-noise ratio and distance between the transmitter and receiver at optimal and coaxial orientations of antenna beams for various propagation scenarios, considering NLOS conditions. In the paper, the influence of the directional antenna use and their direction choices on the channel capacity versus SNR and the distance between the transmitter and receiver is shown.

## 1. Introduction

The effective increase in the capacity of wireless networks in relation to long term evolution networks is one of the main goals in the development of fifth generation (5G) systems. This results from the dynamic increase in the number of users, both people and devices in relation to the Internet of Things [1,2], and the growing demand for the number of provided telecommunications services. On the other hand, network capacity is directly related to the channel capacity (i.e., spectral efficiency) of the individual radio links, which is directly proportional to the bandwidth of the transmitted signals. Thus, increasing the capacity of radio channel conditions is necessary for achieving the above-mentioned aim.

Fifth generation systems will also operate in millimeter-wave bands in addition to the lower frequency ranges of microwaves used so far, i.e., decimeter and centimeter waves [2,3,4,5,6]. Generally, a path loss between a transmitter (TX) and receiver (RX) increases with frequency. The fundamental way to compensate for this increase in attenuation is to increase the energy gain of the antenna system, which is inversely proportional to the width of the radiation pattern. For this reason, in 5G systems, especially for millimeter-wave ranges, directional antennas or multi-antenna systems, including those based on a massive multiple-input-multiple-output (massive-MIMO) technology, will be used [6,7,8]. These types of antenna systems enable beamforming [6,9], which makes a spatial multiplexing technique more effective.

In the transmitting antenna systems, a significant part of the energy is radiated in a specific direction associated with an antenna power pattern direction, i.e., the direction of the main lobe beam. To maximize a received signal power, i.e., minimizing the path loss for the directional link, the direction of the receiving antenna beam should be appropriately selected. Therefore, obtaining the maximum throughput in the directional wireless link requires the implementation of an additional procedure that will ensure the optimal orientation of the antenna beams.

Under line-of-sight (LOS) conditions, the beam directions of the transmitting and receiving antennas are usually directed to each other to maximize the received signal strength. However, in some cases, a beam alignment for LOS conditions is not possible. For example, a base station beam (e.g., as the TX) is oriented in a specific sector direction, while a mobile station (e.g., as the RX) moves along a street canyon, which does not match the transmitting beam direction.

In non-LOS (NLOS) conditions characteristic for urban environments, the effect of a beam misalignment is more visible and important for radio transmission achievement. In this case, the orientation of the antenna beams on each other usually does not guarantee minimizing the path loss. It may result from the occurrence of field obstacles, e.g., buildings, in the TX–RX direction. Therefore, ensuring proper matching of the beam directions of the transmitting and receiving antennas is necessary to maximize the received signal power. It will allow obtaining the optimal capacity in the given propagation conditions.

The main aim of this paper is to evaluate the impact of parameters and the optimal selection of antenna orientation on the radio channel capacity under NLOS conditions. Minimizing the power losses resulting from the mismatch of the antenna beams and optimal choice of their direction is the basis for the radio link quality analysis defined by the SNR. The obtained results have a statistical nature and make it possible to assess the degradation degree of channel capacity, considering the multipath environment propagation. This is the basis for the effectiveness evaluation of the procedures for determining the optimal orientation of both the transmitting and receiving antennas, ensuring maximum capacity under specific propagation conditions. To assess changes in the received signal strength for different antenna beam directions, a geometry-based multi-ellipsoidal propagation model (MPM) [10,11] was used. This model is a three-dimensional (3D) version of the multi-elliptical propagation model which takes into account only the azimuth plane [11]. The MPM considers the influence of the width and directions of beam patterns of the transmitting and receiving antenna systems. It allows modifying the path loss model [12] or received signal strength [13] of the directional link with beam misalignment. As a result, the impact of the transmission parameters of the environment, antenna patterns, and their spatial orientation on the channel capacity can be evaluated. This is the basis for optimizing the antenna beam directions (e.g., in massive-MIMO systems), which ensures the maximization of wireless link capacity under specific environmental conditions. Such an approach to the analyzed problem, which takes into account the influence of a wide range of environmental factors and factors related to the technical parameters of antenna systems, determines the originality of the presented method for the assessment of the channel capacity. The results presented in this paper refer to a strictly determined research scenario, which is defined by the mutual position of the objects (i.e., TX and RX), beam parameters [14], and the transmission properties of the propagation environment described by the power delay profile (PDP) [15]. It is worth highlighting that this methodology used for assessing the channel capacity, which considers the influence of parameters and patterns of antenna beams, is universal and can be applied to various propagation environments and scenarios.

## 2. Related Works

The channel capacity concept and subsequently formulated complete theory of information and its transmission were developed by C.E. Shannon [16,17], based on the earlier works of H. Nyquist and R. Hartley. Currently, this concept of communication channel capacity is called the Shannon–Hartley theorem (or Shannon capacity theorem) and is meant as the theoretical upper bound on the information rate of data that can be transmitted at an arbitrarily low error rate for the set signal-to-noise ratio (SNR). The analyzed channel is classified as an additive white Gaussian noise (AWGN) and memoryless channel. In the wireless link case, it should refer to the LOS and free space propagation conditions, an isotropic antenna, so it does not consider the patterns and parameters of real antennas.

Later works defined capacities for channels with non-dispersive fading and then parallel channels, which also provided an introduction to MIMO channels and spatial diversity systems for dispersive propagation environments. The first works on the ergodic capacity for the MIMO systems took into account Rayleigh fading and different types of MIMO channels [18], e.g., uncorrelated, spatially correlated, double scattering, and keyhole. On the other hand, in the literature, some papers focus on the capacity of channels with Nakagami [19], Rician, Hoyt, or Weibull/log-normal fading [20].

For a few years, two (2D) or 3D geometry-based channel models have been used to evaluate the channel capacity in multipath propagation environments characterized by dispersion in time, frequency, and reception angle domains, e.g., [21]. This research direction shows the influence of the patterns and parameters of antenna systems and the angular dispersion occurring in the real multipath propagation environment on the channel capacity determination. Recently, most of the research on capacity has been devoted to 5G technologies (e.g., [22,23,24,25,26]), networks, and systems [2,9,27,28]. In particular, the analysis of the signal propagation directions from the TX to the RX is crucial for systems based on beamforming and massive-MIMO technologies [25,26]. The use of these technologies in macro and micro-cells, as well as the creation of smaller, i.e., nano-, pico-, and femto-cells (i.e., ultra-dense networks [29]) with the simultaneous use of spectral resources in mm-wave bands [5,30] allows to significantly increase (about 10-fold [29]) not only the spectral efficiency of individual channels and links, but also the capacity of the entire network [2,9,27,28]. This aspect was accurately summarized in [2]: “*… capacity for wireless communication depends on spectral efficiency and bandwidth. It is also related to cell size … Cell sizes are becoming small and physical layer technology is already at the boundary of Shannon capacity …*”.

Antenna beam misalignment in emerging 5G systems, especially in NLOS conditions, is a significant issue from the viewpoint of effective beamforming and tracking procedures, which ensure the achievement of the maximum capacity. Numerous papers, i.a., [31,32,33], presenting both the effects and methods of reducing mismatch, testify to the importance of this topic. The direction mismatch of the transmitting and receiving beams is the reason for the increase in path loss. This fact is demonstrated in [31] by the results of practically performed measurements. The effect of the increase in attenuation is the loss of the received signal power, which results in a significant decrease in the throughput of the directional link. Power losses have a significant impact on reducing the transmission data rate. Examples of solutions that minimize the effects of mismatches for MIMO and hybrid systems, non-orthogonal access systems based on the beamforming technique, are presented in [32,33].

Hence, it may be seen that the analyzed area fits well with the current research trends. In the novel relationship of the channel capacity proposed in Section 3, two impact factors of multipath propagation environment and antenna systems, respectively, were introduced. These factors ensure the appropriate modification of the capacity for the selected single-channel defined by Shannon [16,17]. In the case of MIMO channels, the obtained results should be adequately diversified. This approach is innovative and original in relation to the above-presented methods of channel capacity estimation. In this capacity evaluation, the MPM [11] as a geometry-based channel model, which is based on any PDP, and consider the parameters of antenna beams, was used. It allows for the analysis of the influence of the angular spread of the received signals on the capacity in time-varying channels. On the other hand, the MPM was verified based on empirical results, which provides the basis for the correctness of the presented analyses. Often, the channel capacity is represented in an analytical form and as a graph as a function of SNR, the number of antenna elements in MIMO systems, or environmental parameters (e.g., for different fading distributions). However, from a practical point of view, it is worth illustrating capacity as a function of the TX–RX distance at a given SNR for the reference distance, which was done in this work too.

The remainder of this paper is organized as follows. Section 3 includes the novel approach to express the relationship between the channel capacity and the environmental factors and antenna beam parameters. In Section 4, the MPM description and power angular spectrum (PAS) estimation based on it is shown in short. Next, in Section 5, the impact of the antenna beam directions on the received total power under LOS and NLOS conditions is analyzed. Section 6 depicts the influence of the antenna beam orientation on the radio channel capacity. The results shown in Section 5 and Section 6 were obtained based on simulation studies for the selected spatial scenarios using the MPM and MATLAB environment. Finally, a summary of the paper is contained in Section 7.

## 3. Capacity and Antenna Beam Parameters

The Shannon-Hartley theorem [16,17] introduces the fundamental relationship that describes the relative capacity of the transmission channel, $C_{f},$ in particular, for the radio link

(1) $$C_{f}\left(\mathrm{bit}/s/\mathrm{Hz}\right)=\log_{2}\left(1+SNR\right),$$

where $SNR=P_{f}/P_{n}$ is the ratio of the desired signal power $P_{f}$ to the additive interference power $P_{n}$ in the AWGN form induced in the omnidirectional antenna. In this paper, the above relationship is treated as a reference, which describes the capacity of the radio channel with an omnidirectional antenna system under free-space propagation conditions.

In a multipath propagation environment, especially in NLOS conditions, the level of the desired signal is significantly reduced. It is the cause of the SNR reduction and consequently of the channel capacity. Based on the Friis transmission equation [34], it can be written

(2) $$\begin{array}{ccc} P_{f}\left(D\right)\propto 1/PL_{f}\left(D\right) & \mathrm{and} & P_{m}\left(D\right)\propto 1/PL_{m}\left(D\right), \end{array}$$

where $P_{f}\left(D\right)$ and $P_{m}\left(D\right)$ are the desired signal powers received in free-space and multipath propagation conditions versus distance D between the TX and RX, respectively, while $PL_{f}\left(D\right)$ and $PL_{m}\left(D\right)$ are environmental path loss under free-space and multipath propagation conditions, respectively. For free-space propagation, the path loss in LOS conditions has the form [34]

(3) $$PL_{f}\left(D\right)\left(\mathrm{dB}\right)=20\log_{10}\left(4\pi D/\lambda \right),$$

where $\lambda =c/f_{c}$ and $f_{c}$ are the wavelength and carrier frequency of the transmitted signal, respectively, and c is the lightspeed.

For multipath propagation environments, the path loss may be represented by multiple propagation models. As examples of such models, MiWEBA (Millimetre-Wave Evolution for Back-haul and Access) [35], METIS (Mobile and wireless communications Enablers for the Twenty-twenty Information Society) [36], and 3GPP TR (3rd Generation Partnership Project Technical Report) 38.901 [15] can be pointed out. The radio channel capacity analysis presented in the remainder of the paper is based on the close-in (CI) free-space reference distance path loss model [14], which does not affect the general character of the proposed approach. The CI path loss model is shown in the following form:

(4) $$PL_{m}\left(D\right)\left(\mathrm{dB}\right)=PL_{m}\left(D_{0}\right)+10PLE\log_{10}\left(D/D_{0}\right),$$

where PLE means a path loss exponent (PLE) and $D_{0}$ is the reference distance (for mm-wave, usually $D_{0}=1\;m$). In this case, the propagation conditions are defined by the appropriate selection of PLE values, which were determined based on empirical measurements. For example, these coefficient values for millimeter-waves and selected scenarios are presented in [14]. The main drawback of this path loss determination approach for radio links with narrow-beam antenna patterns is the fact that the measurement data used for the PLE estimation are obtained for strictly determined parameters of the test-bed antennas.

For the purposes of further analysis, the concept of the environmental factor $K_{e}\left(D\right)$ is introduced. It describes the relationship between the received signal powers in a multipath environment $P_{m}\left(D\right)$ and in free-space conditions $P_{f}\left(D\right),$ considering omnidirectional antenna systems. Based on Equation (2), this coefficient can be expressed as

(5) $$K_{e}\left(D\right)=\frac{P_{m}\left(D\right)}{P_{f}\left(D\right)}=\frac{PL_{f}\left(D\right)}{PL_{m}\left(D\right)}.$$

Therefore, assuming the same level of environmental interference (i.e., noise), the channel capacity in the conditions of multipath propagation can be presented in the form

(6) $$C_{m}=\log_{2}\left(1+K_{e}SNR\right).$$

To consider the influence of antenna system parameters on the channel capacity, the antenna system factor is introduced

(7) $$K_{a}\left(D\right)=\frac{P_{s}\left(D\right)}{P_{m}\left(D\right)},$$

where $P_{s}\left(D\right)$ represents the received signal power in the link with the narrow-beam antenna system. This factor describes the relationship between the received signal powers in a multipath propagation environment using narrow-beam antenna systems and omnidirectional antennas.

Introducing the coefficients $K_{e}\left(D\right)$ and $K_{a}\left(D\right)$ makes it possible to determine the functional relationship between the signal powers $P_{s}\left(D\right)$ and $P_{f}\left(D\right)$ received in the links with narrow-beam and omnidirectional antennas in the multipath and free-space propagation environments, respectively,

(8) $$P_{s}\left(D\right)=K_{a}\left(D\right)K_{e}\left(D\right)P_{f}\left(D\right).$$

Hence, the channel capacity $C_{s}$ considering the multipath propagation environment and the narrow-beam antenna system can be expressed by the following formula:

(9) $$C_{s}=\log_{2}\left(1+K_{e}K_{a}SNR\right).$$

Equation (5) shows that the propagation models for free-space and for multipath conditions are the basis for determining the environmental factor $K_{e}\left(D\right).$ On the other hand, the evaluation of the average received power is necessary for determining the antenna system factor, $K_{a}\left(D\right).$ The proposed assessment method of channel capacity uses the MPM to determine $P_{s}\left(D\right)$ in the radio link with the narrow-beam antenna system, considering the multipath propagation conditions. Thanks to this, the influence of both the antenna parameters and the transmission properties of the propagation environment on the channel capacity can be mapped.

In further analysis, as references, Shannon measures of the channel capacities, $C_{f}$ and $C_{d},$ defined for free-space conditions, omnidirectional and directional antennas, respectively, are used. $C_{f}$ is defined by Equation (1), whereas based on the Friis transmission [34], $C_{d}$ considers the change in gains (in linear measure) of the transmitting $G_{T}$ and receiving $G_{R}$ antennas relative to the omnidirectional antennas

(10) $$C_{d}=\log_{2}\left(1+G_{T}G_{R}SNR\right),$$

where SNR is determined for the omnidirectional antennas according to the above definition. In the case of $C_{d},$ the antenna beams are oriented to each other.

## 4. Multi-Elliptical Propagation Model and Power Angular Spectrum Estimation

**Figure 1:**
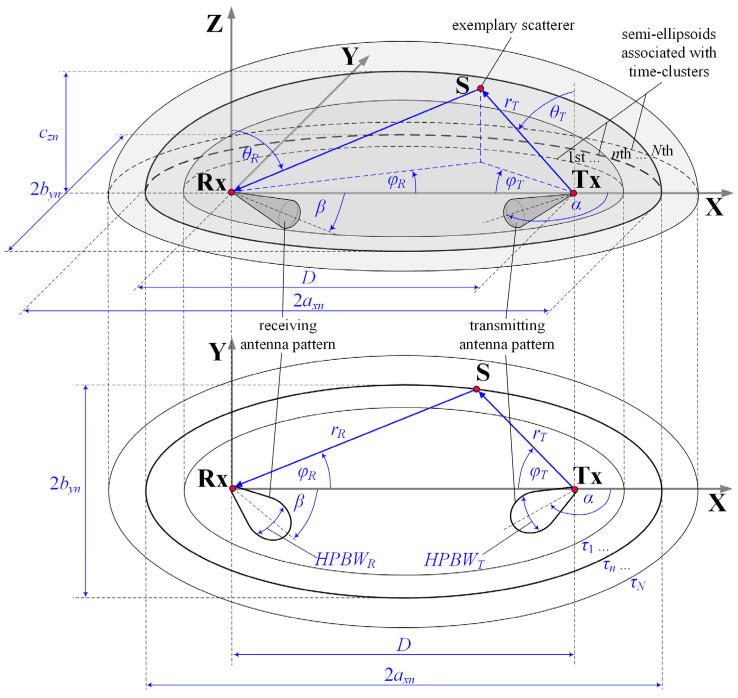
Scattering geometry of MPM.

The MPM provides the estimation of a PAS, $p\left(\theta_{R},\varphi_{R}\right),$ or probability density function (PDF), $f\left(\theta_{R},\varphi_{R}\right),$ of angle of arrival (AOA), $\left(\theta_{R},\varphi_{R}\right),$ where $\theta_{R}$ and $\varphi_{R}$ are the angles in the elevation and azimuth planes, respectively. This model is a 3D geometry-based statistical approach to modeling the spatial scattering of the received signal [10,11]. Figure 1 shows the MPM geometry, i.e., potential scattering areas represented by confocal semi-ellipsoids or ellipses in 3D or 2D model versions, respectively [11]. This geometry results from a PDP defining transmission properties of the analyzed channel. On the other hand, powers defined in the PDP are the basis for determining the power of each propagation path. This solution was firstly used by J.D. Parsons and A.S. Bajwa [37], and next by C. Oestges, V. Erceg, and A.J. Paulraj [38]. In the MPM, it is assumed that the received signal is a sum of delayed components related to the scatterers occurring on the appropriate semi-ellipsoids.

The geometric structure parameters of the MPM are closely related to the transmission properties of the propagation environment described by the PDP. In the case of environments with multipath propagation, the presence of several or a dozen local extremes of the PDP function can be observed. This means that as a result of scattering on obstacles, the electromagnetic wave reaches the RX through various propagation paths. This is the reason why many components of the received signal arrive at the RX with different delays. In practice, the components derived from the single scatterings determine the received signal level. Thus, the semi-ellipsoids can be used to map the most likely positions of the scattering elements. Obviously, the number of ellipsoids is equal to the number of PDP extremes that come from the components of the received signal that form time-clusters with similar delay. If the TX–RX distance is equal to D, then the major $a_{xn}$ and minor $b_{yn},$ $c_{zn}$ half-axes of the *n*th semi-ellipsoid have the form [39]:

(11) $$\begin{array}{ccc} a_{xn}=\frac{1}{2}\left(c\tau_{n}+D\right) & \mathrm{and} & b_{yn}=c_{zn}=\frac{1}{2}\sqrt{c\tau_{n}\left(c\tau_{n}+2D\right)}, \end{array}$$

where $\tau_{n}$ is the delay of the *n*th time-cluster. These delays are determined as arguments of the PDP local extrema. Each of such extremum represents the time-cluster of the reaching propagation paths.

The geometric structure of the MPM has been described in detail in [10,11,39]. The 3D MPM model can be reduced to a 2D multi-elliptical model, in which the propagation phenomena dominate in the azimuth plane [11]. This modeling approach in relation to other geometry-based channel models ensures the minimization of the PAS estimation error as shown in [40]. The efficiency of channel modeling using multi-ellipsoidal geometry is also shown in [41] for the real vehicular-to-infrastructure scenario in the 60 GHz band described in [42].

Estimation of $p\left(\theta_{R},\varphi_{R}\right)$ consists in determining the trajectories of the propagation paths coming from the TX and reaching the RX. These paths consider the multi-ellipsoidal geometry of the scatterer positions. As mentioned, the geometric structure of the MPM maps the potential locations of the scattering elements. Thus, the intersection of the radiated propagation path with the individual semi-ellipses indicates the positions of the scattering elements. Based on the angle of departure (AOD), $\left(\theta_{T},\varphi_{T}\right),$ where $\theta_{T}$ and $\varphi_{T}$ are the angles in the elevation and azimuth planes, respectively, the radial coordinate of the scatterer in the spherical system with the origin in the TX can be determined [39]

(12) $$r_{T}=-\frac{1}{2a}b_{y}^{2}D\sin\theta_{T}\cos\varphi_{T}+\frac{1}{2a}\sqrt{{\left(b_{y}^{2}D\sin\theta_{T}\cos\varphi_{T}\right)}^{2}+4ab_{y}^{2}\left(a_{x}^{2}-\frac{D^{2}}{4}\right)},$$

where $a={\left(b_{y}\sin\theta_{T}\cos\varphi_{T}\right)}^{2}+a_{x}^{2}\left(\cos^{2}\theta_{T}+{\left(\sin\theta_{T}\sin\varphi_{T}\right)}^{2}\right),$ $a_{x}=a_{xn},$ and $b_{y}=b_{yn}.$ Equation (12) is the result of solving the equation system describing the selected semi-ellipsoid and the propagation path line from the TX for the analyzed AOD, $\left(\theta_{T},\varphi_{T}\right)$ [39].

The coordinate transformation involving the translation of the coordinate system origin to the RX allows for the AOA determination of individual propagation paths [39]

(13) $$\theta_{R}=\arctan\frac{\sqrt{{\left(r_{T}\sin\theta_{T}\cos\varphi_{T}+D\right)}^{2}+{\left(r_{T}\sin\theta_{T}\sin\varphi_{T}\right)}^{2}}}{r_{T}\cos\theta_{T}},$$

(14) $$\varphi_{R}=\arctan\frac{r_{T}\sin\theta_{T}\sin\varphi_{T}}{r_{T}\sin\theta_{T}\cos\varphi_{T}+D}.$$

In the simulation procedure for estimating $p\left(\theta_{R},\varphi_{R}\right),$ the normalized radiation pattern of the transmitting antennas, ${\left|g_{T}\left(\theta_{T},\varphi_{T}\right)\right|}^{2},$ is used to generate the AODs, ($\theta_{T},\varphi_{T}$). Since these patterns meet the probability density axioms [43], the PDF of AOD can be written as [39]

(15) $$\begin{array}{c} f_{T}\left(\theta_{T},\varphi_{T}\right)=\frac{1}{4\pi }{\left|g_{T}\left(\theta_{T},\varphi_{T}\right)\right|}^{2}\sin\theta_{T}\\ \begin{array}{cc} \mathrm{for} & \theta_{T}\in \langle 0,\pi /2)\;\mathrm{and}\;\varphi_{T}\in \langle -\pi ,\pi ). \end{array} \end{array}$$

In the MPM, the local scattering phenomenon that occurs in the vicinity of the transmitting and receiving antennas is also taken into account. In this case, the two-dimensional von Mises distribution is used to describe the AOA statistical properties [11,39]

(16) $$\begin{array}{c} f_{0}\left(\theta_{R},\varphi_{R}\right)=C_{0}\frac{\exp\left(\gamma_{\theta }\cos\left(\pi /2-\theta_{R}\right)\right)}{2\pi I_{0}\left(\gamma_{\theta }\right)}\cdot \frac{\exp\left(\gamma_{\varphi }\cos\varphi_{R}\right)}{2\pi I_{0}\left(\gamma_{\varphi }\right)}\\ \begin{array}{cc} \mathrm{for} & \theta_{R}\in \langle 0,\pi /2)\;\mathrm{and}\;\varphi_{R}\in \langle -\pi ,\pi ), \end{array} \end{array}$$

where $\gamma_{\theta }$ and $\gamma_{\varphi }$ define the angular dispersion of the local scattering components in the elevation and azimuth planes, respectively, $I_{0}\left(\cdot \right)$ is the zero-order modified Bessel function of an imaginary argument, and $C_{0}$ represents the normalizing constant such that $\left(C_{0}/2\pi I_{0}\left(\gamma_{\theta }\right)\right)\int_{0}^{\pi /2}\exp\left(\gamma_{\theta }\cos\left(\pi /2-\theta_{R}\right)\right)d\theta_{R}=1.$

In the simulation procedure, the powers of the received signal components that are associated with the individual propagation paths are determined from the PDP. To generate these powers, an exponential distribution whose parameters (i.e., mean values $p_{n}$) are the local extremes of the PDP is adopted

(17) $$f_{p}\left(\tilde{p}\right)=\{\begin{array}{lll} \left(1/p_{n}\right)\exp\left(\tilde{p}/p_{n}\right) & \mathrm{for} & \tilde{p}\geq 0,\\ 0 & \mathrm{for} & \tilde{p}<0, \end{array}$$

where $p_{n}$ is the *n*th local extreme of the PDP that corresponds to the propagation paths from the *n*th semi-ellipsoid.

As a result of the simulation, an ordered set of AOAs, $(\theta_{R},\varphi_{R}),$ and the corresponding powers $\tilde{p}$ are obtained. This set is the basis for the estimation the PAS, $p_{R}\left(\theta_{R},\varphi_{R}\right)$ in the vicinity of the receiving antenna [39]. To obtain the PAS at the output of the receiving antenna, $p\left(\theta_{R},\varphi_{R}\right),$ spatial filtering of $p_{R}\left(\theta_{R},\varphi_{R}\right)$ using the normalized pattern of the receiving antenna, ${\left|g_{R}\left(\theta_{R},\varphi_{R}\right)\right|}^{2},$ should be realized [10,11]. A similar procedure of spatial filtering is described in [15]. A detailed description of the practical implementation of the estimation procedure can be found in [11]. The PAS at the output of the receiving antenna, $p\left(\theta_{R},\varphi_{R}\right),$ are the basis for determining the received power $P_{s}\left(D\right)$ according to the relationship [11]

(18) $$P_{s}=\int_{-\pi }^{\pi }\int_{0}^{\pi /2}p\left(\theta_{R},\varphi_{R}\right)d\theta_{R}d\varphi_{R}=\int_{-\pi }^{\pi }\int_{0}^{\pi /2}p_{R}\left(\theta_{R},\varphi_{R}\right){\left|g_{R}\left(\theta_{R},\varphi_{R}\right)\right|}^{2}d\theta_{R}d\varphi_{R}.$$

Equations (7) and (18) show that the calculation of the antenna system factor, $K_{a}\left(D\right),$ comes down to the determination of $p\left(\theta_{R},\varphi_{R}\right).$

The above description shows that many factors related to electromagnetic wave propagation, which significantly affect the received signal level, are included in the proposed method of the PAS estimation. The transmission properties of the propagation environment characterizing the PDP determine the geometrical structure of the MPM and its spatial parameters. The mapping of the spatial filtration phenomenon by the antenna systems is realized by the utilization of their normalized radiation/reception patterns in the generation procedure of AODs, AOAs, and powers of the propagation paths. This approach to the analyzed problem allows to consider the influence of antenna parameters (i.e., directions of maximum radiation/reception, half-power beamwidths (HPBWs), pattern shape) on the received signal level and, as a result, on the radio channel capacity. This is important in NLOS conditions especially.

## 5. Antenna Orientation and Received Power for LOS/NLOS Conditions

The MPM does not directly provide path loss prediction and only gives us the possibility of assessing the PAS as a normalized function. In practice, many models derived from statistically averaged measurement data can be used to evaluate the path loss. However, these models are defined for the beams directed on each other and selected HPBWs, e.g., [14,44]. Presented in [12,13], the MPM-based methodology provides the modification of the path loss and power balance for different HPBWs and orientations of the antenna beams. A relative power factor, K, is its basis. It represents a relative power for the analyzed beam mismatch and alignment conditions, as follows

(19) $$K\left(\alpha ,\beta ,D\right)\left(\mathrm{dB}\right)=10\log_{10}\frac{P_{s}\left(\alpha ,\beta ,D\right)}{P_{s}\left(\alpha =180{}^{\circ},\beta =0{}^{\circ},D\right)},$$

where $P_{s}\left(D\right)\to P_{s}\left(\alpha ,\beta ,D\right)$ is the received power for the α and β directions of the transmitting and receiving antenna beams (determined with respect to the OX axe in Figure 1), respectively, and the selected distance D. This power is calculated based on Equation (18) and the PAS obtained in the MPM.

### 5.1. Assumptions for Simulation Studies

The evaluation of the power losses resulting from the mismatch of the antenna beams in the directional link and optimal selection of their orientation especially in NLOS conditions, is based on the simulation tests. Additionally, simulation results for LOS conditions to verify the simulation procedure correctness and to show the more complex nature of the propagation phenomenon under NLOS conditions are presented. In the paper, all presented simulation studies were performed based on the MPM implementation prepared in the MATLAB environment.

In simulation studies, a spatial scenario as shown in Figure 1 was analyzed. The adopted scenario may suit communications in microcell between the 5G New Radio gNodeB base station and user equipment operating in the millimeter-wave band. The following assumptions were considered:

- carrier frequency is equal to $f_{c}=28\;\mathrm{GHz};$
- PDPs are based on tapped-delay line (TDL) models from the 3GPP TR 38.901 standard [15], i.e., the TDL-B and TDL-D for NLOS and LOS conditions, respectively; these TDLs are adopted for analyzed $f_{c}$ and rms delay spread, $\sigma_{\tau },$ for so-called the normal-delay profile and urban macro (UMa) scenario, i.e., $\sigma_{\tau }=266\;\mathrm{ns};$
- Rician factor defining the direct path component in the scenario for LOS conditions is appropriate for TDL-D [15], i.e., κ=13.3 dB;
- intensity coefficients of the local scattering components, i.e., the 2D von Mises distribution parameters, are equal to $\gamma_{\theta }=\gamma_{\varphi }=60;$
- distance between the TX and RX is equal to D=50 m;
- beam power patterns consider only the main lobe of the antenna systems. These patterns are modeled by a Gaussian model [43] for the appropriate beam parameters, i.e., HPBWs and gain.
- HPBWs of the transmitting and receiving antennas are the same in the azimuth and elevation planes, i.e., $HPBW_{T\theta ,R\theta }=HPBW_{T\varphi ,R\varphi }=10{}^{\circ}$ based on the real antenna parameters used in [14,44];
- gains of the transmitting and receiving antennas are calculated based on the following formula [45,46]:
  (20) $$G_{T,R}=\frac{41253\eta }{HPBW_{T\theta ,R\theta }HPBW_{T\varphi ,T\varphi }},$$
  where η=0.7 is a typical average antenna efficiency. By extension, the gains are equal to $G_{T}=G_{R}=24.6\;\mathrm{dBi}$ for the transmitting and receiving antennas, respectively.
- Low heights of the transmitting (7 m) and receiving (1.5 m) antennas are based on measurement scenarios [14];
- beam alignment is defined for α=180° and β=0° (see Figure 1);
- analyzed ranges of beam directions are as follows: 90°≤α≤270° and −90°≤β≤90°;
- steps of changing the antenna directions in simulation studies are Δα=Δβ=1°;
- to obtain average statistical results in the MPM, L=10 paths are generated at the TX for each time-cluster (semi-ellipsoid). On the other hand, M=360 Monte-Carlo simulations were run for each analyzed scenario; in this case, the average resolution of generating the AODs is about 0.1°.

In accordance with the purpose of simulation tests, the mismatch effects of directions between the transmitting and receiving beams are presented. As a measure of the power loss of the received signal, which results from the beam misalignment, the factor K(α,β,D)→K(α,β) defined by Equation (19) was used.

These studies relied on the Gaussian model [43] for the main lobe of the antenna pattern. However, it should be highlighted that the MPM may consider any pattern shape. For example, in [47,48], the actual pattern of 5G New Radio gNodeB base station antenna system based on the massive-MIMO technology was implemented in the MPM for downlink and uplink inter-beam interference analysis.

### 5.2. LOS Conditions

**Figure 2:**
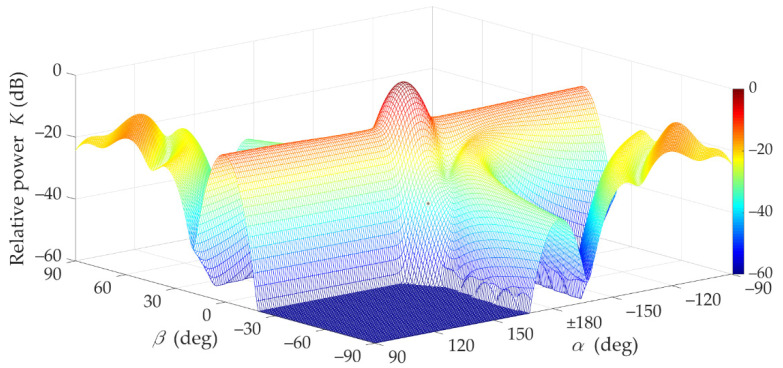
Relative power factor *K*(*α*,*β*) versus *α* and *β* directions of transmitting and receiving beams under LOS conditions (3D graph).

**Figure 3:**
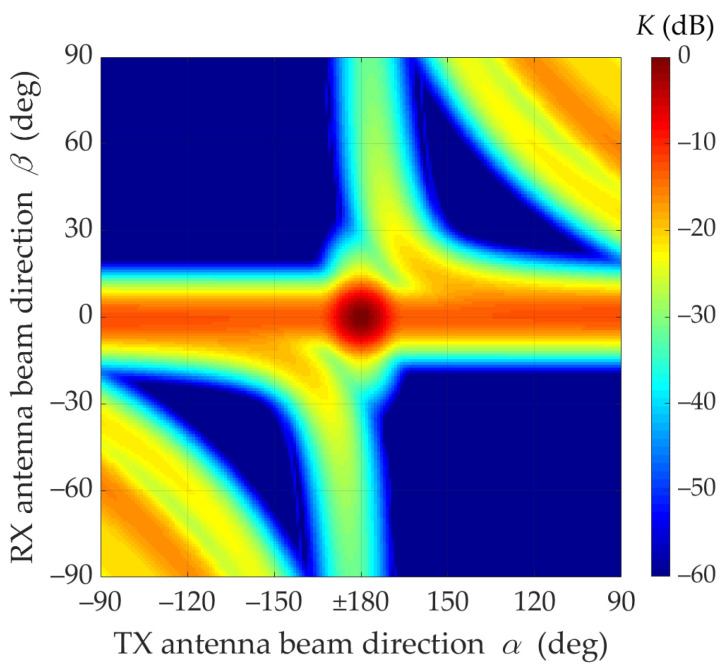
Relative power factor *K*(*α*,*β*) versus *α* and *β* directions of transmitting and receiving beams under LOS conditions (2D graph).

First, the effects of beam direction mismatch for LOS conditions (i.e., for TDL-D [15]) are presented. In Figure 2 and Figure 3, K(α,β) as a function of α and β directions of the transmitting and receiving antenna beams is illustrated.

**Figure 4:**
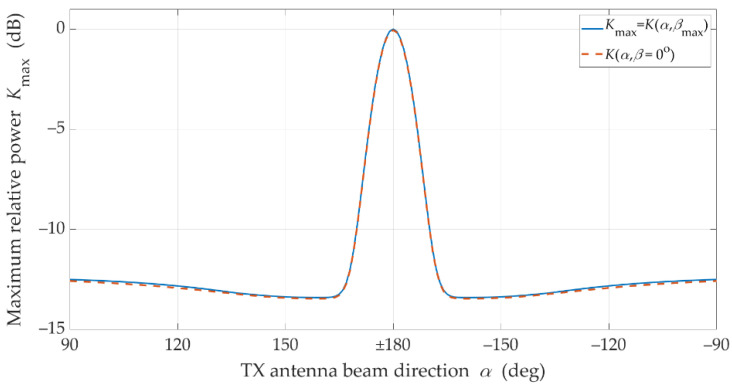
Received signal power losses due to mismatch of transmitting beam direction under LOS conditions.

These graphs clearly show that when the beams are directed at each other, a dominant received power is obtained. In addition, it may be seen that if the transmitting beam is not directed at the RX (i.e., α≠±180°), the occurring power losses can be partially compensated by the optimal selection of the receiving beam direction β. The compensation efficiency of the receiving beam direction is depicted in Figure 3. Additionally, in Figure 4, the power losses that occur while maintaining a constant receiving angle β=0° (red dashed line) are shown.

In the analyzed LOS conditions, the Rician factor is equal to 13.3 dB. This means that the first time-cluster in the PDP is dominant. Thus, the direct path (i.e., $\tau_{0}=0$) significantly determines the received power for beam misalignment as well, which results from the Friis equation [34]. Figure 2 and Figure 3 show that the maximum power is obtained for beam alignment, which is obvious. The direction of the receiving antenna has a decisive influence on the power level. Despite the direction changes of the transmitting antenna, the extremum power is ensured when the RX antenna is pointed at the TX.

**Figure 5:**
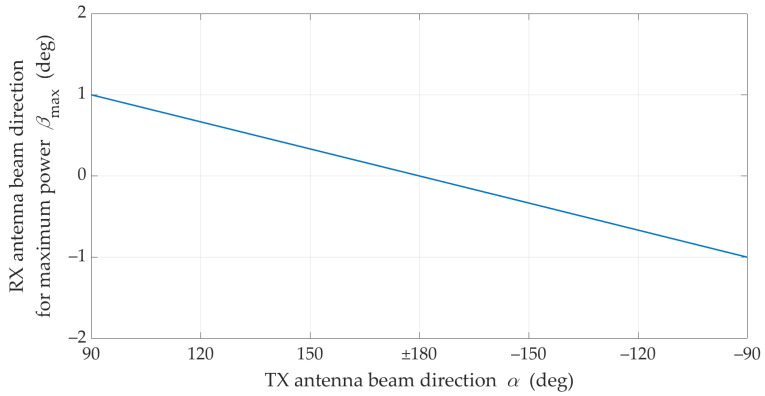
Optimal reception angle for different transmitting beam direction under LOS conditions.

For individual α, the graph of the optimal reception angle (i.e., the optimal direction of the receiving beam), $\beta_{\max},$ which ensures the maximization of the received signal power, i.e.,

(21) $$K_{\max}=K\left(\alpha ,\beta_{\max}\right)=\underset{-90{}^{\circ}\leq \beta \leq 90{}^{\circ}}{\max}K\left(\alpha ,\beta \right),$$

is presented in Figure 5.

The charts in Figure 4 and Figure 5 prove the obvious conclusion graphically. For LOS conditions, the optimal direction of the receiving beam is generally constant and equal $\beta_{\max}≅0{}^{\circ}.$ Practically, it means that in this case it has no way of compensating the effects of mismatching the direction of the transmitting beam by proper selection of the receiving beam direction. This is due to the presence of the delayed components (i.e., time-clustes for $\tau_{n}>0$) in addition to the dominant direct path occurring under LOS conditions. In free space conditions, the direct path appears only. In this case, the graph in Figure 5 would have a constant value for $\beta_{\max}=0{}^{\circ}.$

### 5.3. NLOS Conditions

**Figure 6:**
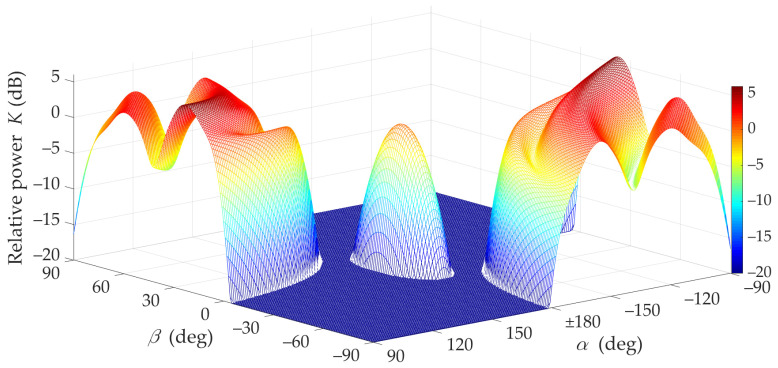
Relative power factor *K*(*α*,*β*) versus *α* and *β* directions of transmitting and receiving beams under NLOS conditions (3D graph).

**Figure 7:**
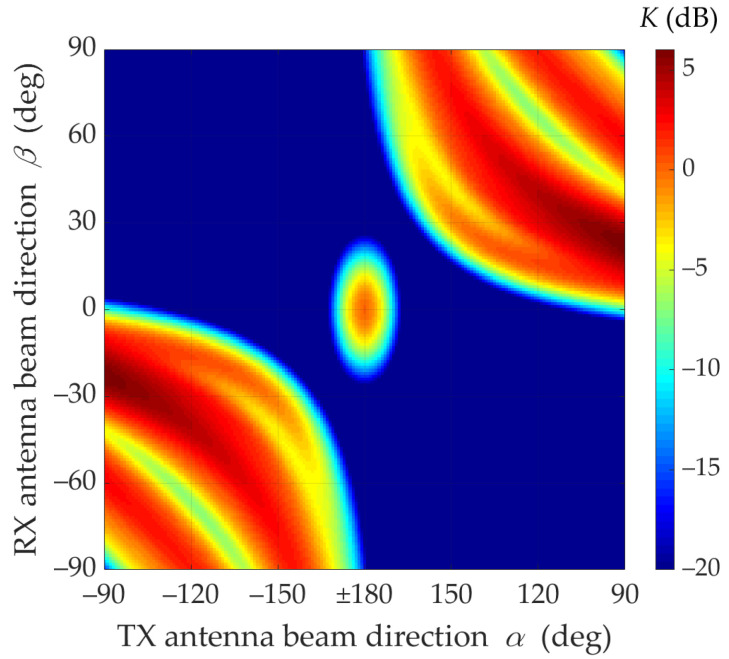
Relative power factor *K*(*α*,*β*) versus *α* and *β* directions of transmitting and receiving beams under NLOS conditions (2D graph).

Under NLOS conditions (i.e., for TDL-B [15]), the multipath propagation phenomenon makes it necessary to search for optimal α and β directions of the transmitting and receiving antenna beams, which will ensure the maximization of the received signal level. Figure 6 and Figure 7 illustrate the mismatch effects between the TX and RX beam directions.

**Figure 8:**
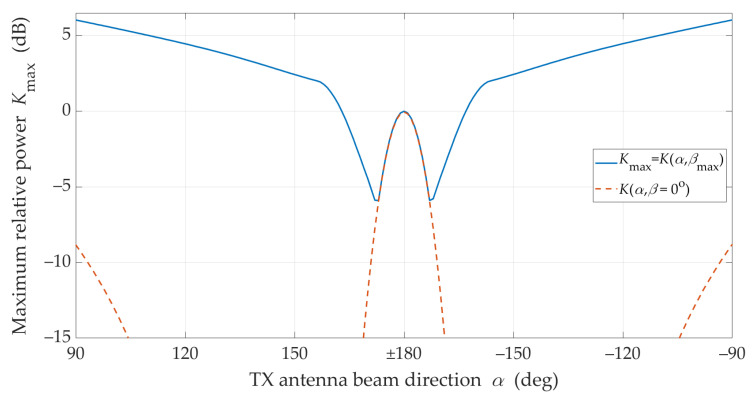
Compensation effectiveness for beam misalignment under NLOS conditions.

The graphs presented in Figure 8 show that in NLOS conditions for a limited range of changes |α|<90° and for assumed parameters of antennas and propagation environments, a 6 dB increase in the received power can be achieved by optimal selection of the TX and RX beam angles. This means that under these propagation conditions the mutual coaxiality of the beams does not provide the highest received signal level.

Furthermore, Figure 8 shows that for the analyzed scenario, in the absence of coaxiality of the transmitting beam direction, which exceeds ±15°, the optimal selection of the receiving beam direction provides the possibility of increasing the received power in relation to the beam alignment. Therefore, in systems using the massive-MIMO and beamforming technologies, the optimal selection of the antenna beam directions should be provided.

**Figure 9:**
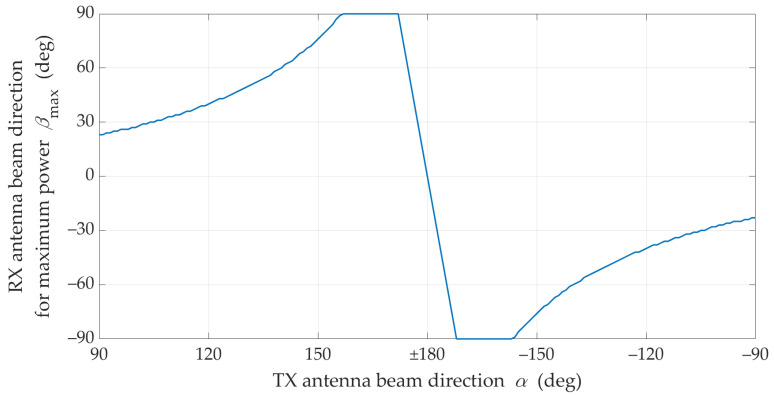
Optimal reception angle for different transmitting beam direction under NLOS conditions.

The statistical evaluation of an optimal direction of the receiving beam corresponding to the transmitting beam misalignment is presented in Figure 9.

Figure 7 and Figure 8 show that under NLOS conditions, the received signal obtains the statistically highest power level for the TX beam direction equal to α=±90°. However, in this case, the beam direction of the receiving antenna to achieve this power level should be equal $\beta_{\max}≅23{}^{\circ}.$

The lack of the direct path (i.e., the Rician factor equal to 0) under NLOS conditions is the principal cause of the difference in results in relation to those obtained for LOS conditions. For NLOS conditions, the majority of the received power comes from the delayed components scattered on the semi-ellipsoids. Therefore, the global maximum of the received power does not appear for α=180° and β=0° (see Figure 6, Figure 7 and Figure 8).

## 6. Antenna Orientation and Radio Channel Capacity

The results presented in Section 5 show that under NLOS propagation conditions, to obtain the maximum received signal level on the radio link with directional antennas, it is necessary to determine the optimal orientation of the antenna beams. Of course, in LOS conditions, this problem does not arise since the direction determined by the TX and RX positions is the direction of the maximum received signal level. In this section, an assessment of the impact of both environmental transmission properties and antenna orientations on the channel capacity under NLOS conditions is presented. As reference data, the Shannon channel capacities $C_{f}$ and $C_{d}$ with the omnidirectional and directional antenna systems under free-space propagation conditions, respectively, were assumed.

The assessment of the received signal level, and consequently the SNR, which directly determines the channel capacity, is based on the MPM use in the simulation test procedure, considering antennas with narrow radiation/reception beams. These studies are carried out for the assumptions described in Section 5, taking into account the changes in the distance D. The transmission properties of the propagation environment are reflected in the adopted CI path loss model [14] described by Equation (4).

**Figure 10:**
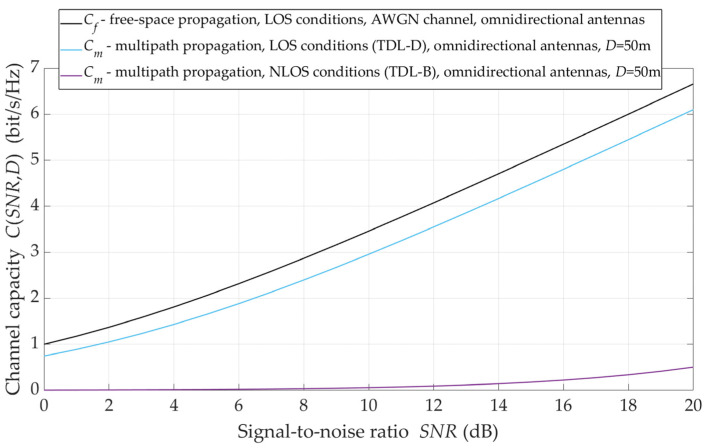
Capacity versus SNR for omnidirectional antenna pattern and different propagation conditions.

This influence maps the channel capacity variation with the omnidirectional antenna systems as a function of the reference SNR, which corresponds to free-space propagation conditions. In this case, the SNR is directly proportional to the emitted signal level. For free-space LOS (blue line), multipath LOS (red line), and multipath NLOS (black line) propagation conditions, the graphs of capacity as an SNR function are shown in Figure 10.

The obtained results are a graphical representation of Equations (1) and (6). For multipath propagation, the $K_{e}$ coefficient is determined based on Equations (4) and (5), where LOS and NLOS propagation conditions are determined by the PLE values equal to PLE=2.1 and PLE=3.4, respectively [14]. These graphs show that the radio channel capacity under LOS conditions in relation to free-space is only 0.5 bit/s/Hz less. On the other hand, the NLOS conditions significantly reduce the channel capacity even several times.

**Figure 11:**
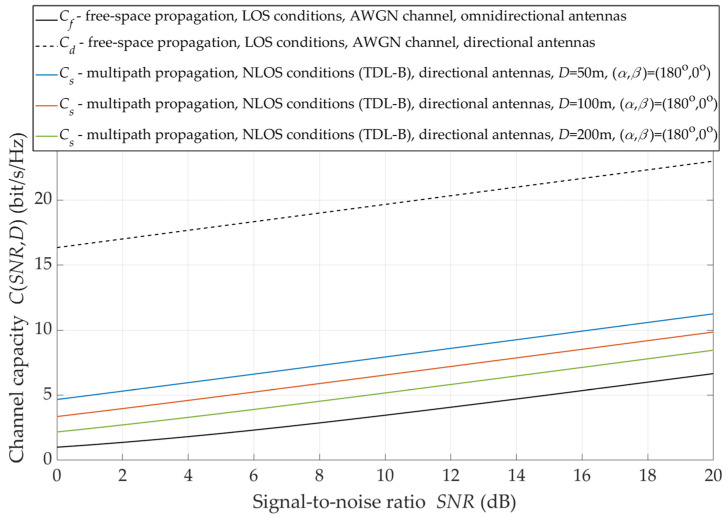
Capacity versus SNR for directional antenna pattern and under NLOS conditions.

The use of antenna systems with narrow-beam radiation/reception patterns is one way to minimize the negative effects of multipath propagation under NLOS conditions. The effects of using spatially selective antenna systems are shown in Figure 11.

For LOS conditions, the alignment of the transmitting and receiving antenna beams (i.e., beams oriented to each other) provides statistically multiple increases in the radio channel capacity. Of course, this increase depends on the gains of the antennas. For the analyzed radio link with the directional antennas whose gains are equal to $G_{T}=G_{R}=24.6\;\mathrm{dBi},$ the capacity is higher by 1.5 ÷ 3.5 bit/s/Hz in relation to the radio link with the omnidirectional antenna systems and free-space propagation conditions. The analysis results presented in Figure 11 show that the radio channel capacity also depends on the distance between the TX and RX. The double distance reduction increases the radio channel capacity by about 1.5 bit/s/Hz in the whole analyzed range of SNR variability.

**Figure 12:**
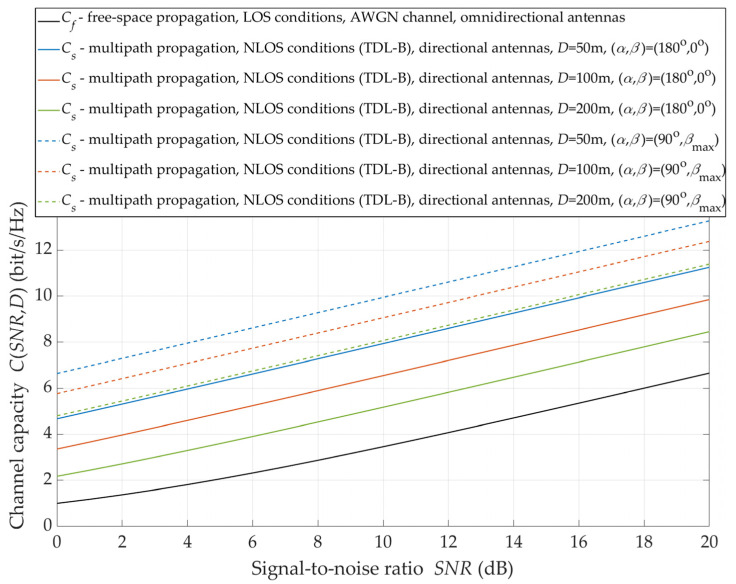
Capacity versus SNR for *β*_max_ under NLOS conditions.

Under NLOS conditions, the alignment of the transmitting and receiving antenna beams does not provide to achieve the maximum received power. Therefore, under these propagation conditions, the massive-MIMO system should supply a beam steering mechanism to the direction of the maximum level of the received signal. The justification for the application of such a solution is illustrated in Figure 12.

It can see that the use of the direction of the maximum signal level ensures an additional increase in the capacity by 2 ÷ 3 bit/s/Hz. The increment increases as the TX–RX distance is greater. For D=50 m, the selection of the direction of the maximum level ensures a statistical increase in the channel capacity by about 2 bit/s/Hz, while for D=200 m, this increase is reduced to 3 bit/s/Hz.

**Figure 13:**
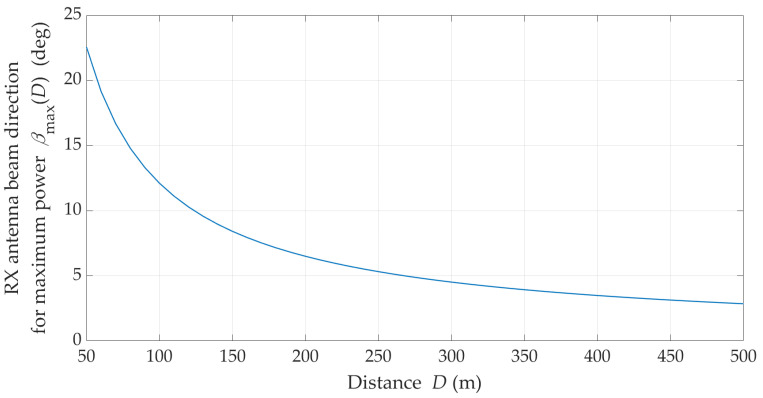
Statistical value of *β*_max_ versus TX–RX distance under NLOS conditions.

The channel capacity change versus the distance between the TX and RX, considering the stability of the emitted power (i.e., its constant value), makes it possible to practically assess the reception effectiveness for $\beta_{\max}.$ However, changing the TX/RX position makes it necessary to search for the reception direction of the maximum signal level. Changes in $\beta_{\max}$ as a function of D for α=90° are shown in Figure 13.

The presented result of $\beta_{\max}$ versus D has a statistical nature because the simulation studies using the MPM are based on the statistical transmission characteristics of the channel (i.e., TDLs from the 3GPP standard [15]). On the other hand, this statistical nature arises from averaging the results over several simulation cycles. As can be seen, as the distance increases, the reception directions for the maximum signal level converge to $\beta_{\max}\to 0{}^{\circ}.$

**Figure 14:**
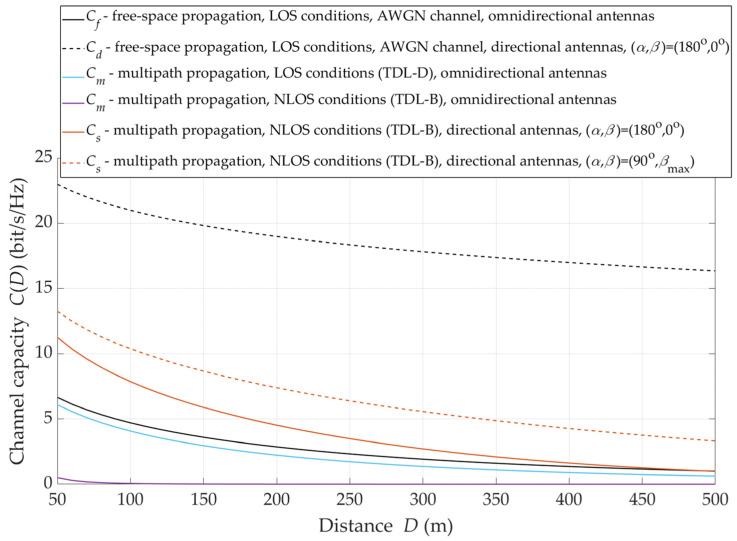
Channel capacity versus TX–RX distance for straight and optimal directions of antenna beams under NLOS conditions.

Determining the radio channel capacity for optimal receiving beam direction makes it possible to evaluate the system effectiveness for selecting the signal reception direction. The comparison of the capacity change for the straight (i.e., α=180° and β=0°) and optimal (i.e., α=90° and $\beta =\beta_{\max}$) directions of the antenna beams is shown in Figure 14.

The exemplary graphs are obtained assuming that, at D=50 m, the level of the received signal provides the SNR=20 dB. It is obvious that as the TX–RX distance increases, the level of the desired signal decreases. Thus, the radio channel capacity decreases. In the case of the optimal direction of the antenna beams, a six-fold increase in the distance causes only about a 2.5-fold reduction in the capacity. On the other hand, with the increase in the distance, maintaining the straight direction results in a 4.4-fold decrease in the capacity. This shows that the use of steering and selection of the optimal beam direction in the antenna system (e.g., massive-MIMO) mitigate the degrading effect of distance on the radio channel capacity.

## 7. Conclusions

This paper focuses on the influence assessment of the antenna system parameters, with particular emphasis on their orientation, on the radio channel capacity under NLOS propagation conditions. The need to take up such topics is related to implementing new technologies in antenna systems with beamforming and tracking technologies, e.g., massive-MIMO. The performed evaluation has a statistical nature and is based on simulation studies. In this case, the MPM was used to map the effects of propagation phenomena. The obtained results show that under NLOS conditions, it is desirable to use directional antennas as this provides significant compensation for signal attenuation. The effect of this is as follows:

- a dozen or so times increase in the radio channel capacity compared to the omnidirectional antenna;
- the direction selection of the maximum received signal level increase by about 2 bit/s/Hz the channel capacity regardless of the TX–RX distance;
- the control system for selecting the reception direction of the maximum signal level increases the capacity of the link, and its efficiency increases with increasing distance.

The issues presented in this article are of significant practical importance. The proposed procedure provides a quantitative assessment of the efficiency of using beam-steering antenna systems under NLOS conditions. The use of the geometry-based MPM in the simulation tests presents the possibility to consider not only the parameters and patterns of the antenna system, but also the type of propagation environment. Thanks to this, the method of analyzing the capacity of the directional radio links enables the evaluation of the spatial range of the implementation of complex telecommunication services. This is important in the process of planning the area covered by base stations. This determines the originality of the radio channel capacity analysis method described in this paper in comparison to the methods presented so far in the literature.

In the future, the authors plan to conduct empirical research for selected scenarios that will allow to verify the approach presented in this paper. Additionally, the authors also want to compare the impact of utilizing a simplified antenna pattern (i.e., Gaussian model for the main lobe) with a real pattern (i.e., considering the side-lobes) on various parameters of the directional radio link, including throughput, interference, energy balance, and angular spread.

## Data Availability

The data presented in this study are available on request from the corresponding author. The data are not publicly available due to project restrictions.

## Abbreviations

2D	two dimensional
3D	three dimensional
3GPP	3rd Generation Partnership Project
5G	fifth-generation
AOA	angle of arrival
AOD	angle of departure
AWGN	additive white Gaussian noise
CI	close-in free-space reference distance (path loss model)
HPBW	half-power beamwidth
LOS	line-of-sight
METIS	Mobile and wireless communications Enablers for the Twenty-twenty Information Society
MIMO	multiple-input-multiple-output
MiWEBA	Millimetre-Wave Evolution for Back-haul and Access
MPM	multi-elliptical propagation model
NLOS	non-line-of-sight
PAS	power angular spectrum
PDF	probability density function
PDP	power delay profile
PL	path loss
PLE	path loss exponent
RX	receiver
SNR	signal-to-noise ratio
TDL	tapped-delay line
TR	technical report
TX	transmitter
UMa	urban macro
